# Implementation of a patient centred medical home (PCMH) initiative in general practices in New South Wales, Australia

**DOI:** 10.1186/s12875-021-01485-x

**Published:** 2021-06-21

**Authors:** Christine Metusela, Bridget Dijkmans-Hadley, Judy Mullan, Andrew Gow, Andrew Bonney

**Affiliations:** 1 General Practice Academic Unit, School of Medicine, University of Wollongong, Wollongong, Australia; 2 The Royal Australian College of General Practitioners, East Melbourne, Australia; 3 South Eastern New South Wales Primary Health Network, Wollongong, Australia

## Abstract

**Background:**

With an ageing population and an increase in chronic disease burden in Australia, Patient Centred Medical Home (PCMH) models of care have been identified as potential options for primary care reform and improving health care outcomes. Adoption of PCMH models are not well described outside of North America. We examined the experiences of seven general practices in an Australian setting that implemented projects aligned with PCMH values and goals supported by their local Primary Health Network (PHN).

**Method:**

Qualitative and quantitative data were collected over a twelve month period, including semi-structured interviews, participant observation, and practice data to present a detailed examination of a subject of study; the implementation of PCMH projects in seven general practices. We conducted 49 interviews (24 pre and 25 post) with general practitioners, practice managers, practice nurses and PHN staff. Framework analysis deploying the domains of a logic model was used to synthesis and analyse the data.

**Results:**

Facilitators in implementing successful, sustainable change included the capacity and willingness of practices to undertake change; whole of practice engagement with a shared vision towards PCMH change; engaged leadership; training and support; and structures and processes required to provide team-based, data driven care. Barriers to implementation included change fatigue, challenges of continued engaged leadership and insufficient time to implement PCMH change.

**Conclusions:**

Our study examined the experiences of implementing PCMH initiatives in an Australian general practice setting, describing facilitators and barriers to PCMH change. Our findings provide guidance for PHNs and practices within Australia, as well as general practice settings internationally, that are interested in undertaking similar quality improvement projects.

**Supplementary Information:**

The online version contains supplementary material available at 10.1186/s12875-021-01485-x.

## Introduction

In Australia, as internationally, the ageing population and associated increase in the burden of chronic illness is well recognised [1–3]. This is reflected in general practice in the caseloads of practitioners where currently over 40% of all consultations address chronic illness [4, 5]. There is a pressing need therefore for high-performing primary care that focuses on preventive health and the management of chronic disease [6]. Understandably, there is significant health policy interest in structural reform to enable the health system to best meet the needs of an ageing population and increasing prevalence of chronic disease [7]. Current examples of reform include pay for performance programs such as the UK Quality and Outcomes Framework (QOF) and the Australian Practice Incentives Program Quality Improvement (PIP QI) aimed at rewarding general practice for delivering good quality care [8, 9]. Expanding the medical workforce and enhancing the roles of nursing staff and other health care professionals in a patient centred, team-based care environment, are also elements of practice reorganisation that have been recommended in an Australian setting [10, 11].

Patient-Centred Medical Home (PCMH) models of care have been active and evolving in the United States for over a decade [12]. The PCMH concept is based on restructuring patient care; enhancing patient experience of care, improving efficiency and use of information technology, thereby improving health outcomes and ultimately, reducing emergency room visits, hospital admissions and overall health care costs [13–15]. The PCMH model has been described more recently, in relation to ten “building blocks” that characterise high-performing primary care, with foundational elements of engaged leadership, data-driven improvement, empanelment (linking patients to a care team), and team-based care [16]. PCMH models are also aligned to the Quadruple Aim [17]; enhancing patient and provider experience, improving the health of the population and reducing health related costs.

Following positive outcomes in primary care reorganisation in North America [12, 18], Canada [19] and New Zealand [20, 21], the Royal Australian College of General Practitioners (RACGP) has identified the PCMH model of care as a potential vehicle for enhancing continuity and coordination of care to improve outcomes and maintain sustainability in the Australian primary care system [3, 22]. In 2017, the Australian government commenced the Health Care Homes (HCH) trial which has a particular focus on providing services to patients with chronic and complex conditions [23]. The HCH model is based on PCMH components and aligns with recommendations from various national bodies including the Department of Health [24], the Primary Health Care Advisory Group [2], and the National Hospitals and Health Reform Commission [7]. The model is underpinned by funding reform that provides tiered, bundled payments allocated according to individual patient complexity and is intended to replace components of the current fee-for-service arrangement [23, 25]. Approximately 200 general practitioner (GP) practices and Aboriginal Community Controlled Health Services (ACCHS) in Australia are involved in the HCH trial which runs until 2021 [23].

General practitioners are the usual first point of contact for patients accessing health care in Australia [26]. Approximately 95% of general practice income is obtained from fee-for-service payments through the federal government health insurance scheme, Medicare [27]. Under this scheme, approximately 83% of visits are ‘bulk-billed’, or without charge to the patient [27, 28]. Primary Health Networks (PHNs), independent organisations funded by the Australian government, are central to the government’s care reforms in improving the quality of primary care in Australia [29, 30]. The South Eastern New South Wales PHN (SENSW PHN), although not a HCH trial site, identified that a project focused on improving efficiency, effectiveness and co-ordination of locally-based primary care services would be one way to support the Government’s primary care reform agenda. The PHN commissioned a broad stakeholder consultation across its footprint which subsequently led to the development of a logic model as an implementation framework for PCMH-related practice redesign (see Additional file 1). They have chosen this approach because of the evidence that logic models are useful planning and evaluation tools for monitoring and evaluating implementations, including those used in primary care [31]. The PHN then called for expressions of interest from local general practices to develop and implement PCMH-related projects with PHN support (including funding of up to $70,000). These projects were run between June 2017 and June 2018.

There is accumulating evidence that a PCMH approach is associated with improved patient experience of care, reduced staff burn out, and fewer hospitalisations compared with traditional models of primary care [32–34]. The majority of research regarding PCMH change emanates from North America and there are limited data reporting PCMH change efforts in Australia [35]. International experience suggests that transitioning to a PCMH model requires substantial transformational change, one that involves considerable time and costs [36–38]. In this paper we present our findings on the experiences of the South Eastern New South Wales PHN and seven general practices in implementing PCMH-related projects, with a view to informing similar practice change both nationally and internationally.

## Methods

A multi-site case study methodology was utilised as the primary research approach. The research design, based on a pragmatic approach [39], was informed by responsive evaluation, which observes the rationale for a particular program, in this case PCMH projects, and responds to emerging and preconceived issues in the implementation process [40]. Furthermore, it also allows for both qualitative and quantitative data analyses and places importance on understanding people and programs in context [41, 42]. The aforementioned logic model (Additional file 1) was used as an implementation framework. Aligned with Nilsen (2015) [43], rather than specifying mechanisms of change, the framework consisted of various descriptive categories perceived to influence implementation outcomes.

### Participants and data collection

We used a mixed methods approach collecting and synthesising qualitative and quantitative data in this study. Multiple data were collected over the twelve month period of project implementation, including semi-structured interviews, participant observation and aggregate project outcome data.

Eight practices were invited by the PHN to participate in an evaluation of the PCMH projects. Seven consented and one declined due to extenuating circumstances beyond their control. Once practice consent was received, the research team contacted the practices to arrange visits to each participating practice. Appropriate observation points for the evaluation were confirmed and potential interview participants purposively identified by the practice. Purposively selected PHN staff were also invited directly by phone or email to participate in the study with consenting participants then contacted to be interviewed.

### Semi-structured interviews

To provide a variety of perspectives and experiences of implementing the PCMH projects, semi-structured interviews were conducted with a range of key stakeholders, including general practitioners, practice nurses and practice managers, allied health professionals and PHN staff (see Table 1). Refer to Additional file 2 for the interview topic guides.

**Table 1:** Participant sample

Participant type	Number of participants
Primary Health Network (PHN) Staff	9
General practice staff:	
• Practice Nurse (PN)	5
• Practice Manager (PM)	7
• General Practitioner (GP)	5
• Pharmacist (PH)	1
**Total**	**27**

In consultation with the project Advisory Group that was formed to provide project guidance (consisting of two PHN senior managers, UOW research team consisting of two senior academics and the project officer), and informed by the literature [16, 18], we designed interview guides that were minimally adapted for the different projects and participants groups. The semi-structured interviews investigated the implementation process and attainment of goals of the individual projects through participant experiences and perspectives.

Semi-structured interviews were arranged with consenting participants and conducted by one of the authors (BDH), either face-to-face or by telephone according to participant preference. Interviews were audio-recorded and transcribed verbatim. Interview transcripts were de-identified and participants assigned de-identified labels. Transcripts were checked for accuracy and offered to participants for review.

### Participant observation

During the twelve month implementation period, in-practice participant observation was conducted by BDH to explore the concerns, successes and challenges of those involved in the projects. Data were collected at all seven practices and with PHN staff through observations, researcher field notes, meeting minutes from telephone project follow-ups, and informal discussions. Approximately 25 h of observation took place, with time frames varying from between 15 min and one hour (37.5 min on average). This included an initial in-practice visit at the beginning of the project with each practice and also 20 further points of contact, dependent on the needs of the individual practices (see Table 2). Observations were also carried out at six sessions with the PHN and at six Advisory Group meetings.

**Table 2:** Observation activities with practices

Sites	Observation activities
Practice 1	Observation at initial in-practice evaluation visit (*n* = 1) Follow-up sessions with practice (*n* = 3) Observation at a practice nurse workshop (*n* = 1)
Practice 2	Observation at initial in-practice evaluation visit (*n* = 1) Follow-up sessions with practice (*n* = 2)
Practice 3	Observation at initial in-practice evaluation visit (*n* = 1) Follow-up sessions with practice (*n* = 3)
Practice 4	Observation at initial in-practice evaluation visit (*n* = 1) Follow-up sessions with practice (*n* = 2) Observation of waiting room prior to clinic set-up, during clinic set-up and informal feedback of clinic from staff or patients (*n* = 1)
Practice 5	Observation at initial in-practice evaluation visit (*n* = 1) Follow-up sessions with practice (*n* = 3) Observation of patient education session (*n* = 1)
Practice 6	Observation at initial in-practice evaluation visit (*n* = 1) Follow-up sessions with practice (*n* = 2)
Practice 7	Observation at initial in-practice evaluation visit (*n* = 1) Follow-up sessions with practice (*n* = 2)

### Aggregate practice data

Patient data related to the individual projects were collected by practice staff within the seven practices. At the end of the implementation period, each practice provided the research team aggregate de-identified data from their pre- and post-project audits. These aggregate data represent an evaluation to ‘a point in time’ of the PCMH projects (refer to Additional file 3 for a description of the audit tools).

### Analysis

**Fig. 1:**
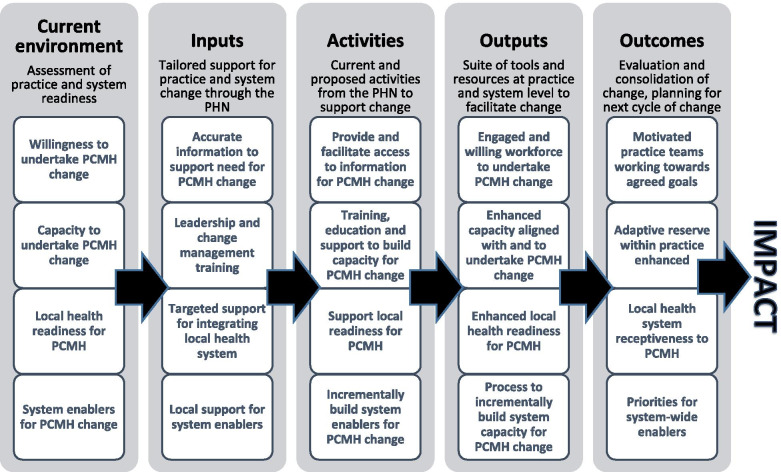
PCMH logic model

Framework analysis supports a comprehensive and descriptive overview of an extensive data set and is congruent with our use of responsive evaluation methodology [44]. We used a framework analysis approach applying a logic model as an implementation framework to analyse our data. The framework of the logic model (see Fig. 1) enabled us to describe the processes of implementing the PCMH projects, the experiences around these, their perceived value, and facilitators and barriers in achieving the desired project outcomes.

N-Vivo 11 ® software was used to help organise the data set. Two members of the research team independently performed the initial coding of the data using the logic model framework, consistent with best practice guidelines [45]. These researchers then met to discuss and review the initial coding. One of the researchers (BDH) then coded the remainder of the data set, with ongoing input from the research team. Upon completion of coding, the research team met to review and discuss the completed coding matrix and to ensure the framework was inclusive of the data. The research team engaged in reflexivity throughout the analysis, being aware their experiences and socio-cultural backgrounds helped shape the research findings [45]. The research received ethics approval from the University of Wollongong Human Research Ethics Committee (REF 2017/236).

## Results

Seven practices and 27 participants (18 general practice staff and 9 PHN staff) were directly involved in the study (refer to Table 1), with most participants engaged at differing time points during the study. A total of 49 semi-structured interviews were conducted between December 2017 and June 2018, with 22 participants participating in two interviews and five participating in only one interview. Participant observation data (described using the phrase “it was observed”) and aggregate project outcome data were also collected at all seven practices.

The individual projects undertaken by each of the practices are described in Table 3. Three of the PCMH-based projects focused on mental health interventions, while the remainder targeted chronic conditions (osteoporosis, respiratory illness and diabetes) and a consultant pharmacist within the general practice.

**Table 3:** Description of the PCMH projects

Practices	Description of PCMH Project
P1	Expansion of the Teen Clinic program (a nurse led drop-in service for young people in rural towns) to four general practices in the region
P2	Establishing a Teen Clinic within the practice with a focus on improving access to mental health services for young people in rural areas
P3	Psychosocial program to support the mental health of refugee patients with activities such as workshops, developing strength and resilience cards, reflective patient journals and walking groups
P4	Osteoporosis education program targeting patients aged over 70, consisting of bone health group sessions with patients, their GPs and allied health
P5	Respiratory disease program with an Integrated Care approach for patients with Asthma and COPD involving a clinical nurse consultant and a GP integrated appointment
P6	Integration of a consultant pharmacist within the practice to discuss medication adherence with patients and to check that medication records are up to date
P7	Coordinated diabetes management care plan focussing on a practice nurse led review of diabetes patients aged between 50 and 60 living in low socio-economic areas

Characteristics of the participating practices and demographics of the practice locations are provided in Table 4 below. Four of the seven practices had more than five GPs with four practices located in inner regional areas (RA2) and three in major cities (RA1). Most practices were situated in areas of disadvantage, with only one practice located in an area with a Socio-economic Indexes for Areas (SEIFA) [46] decile greater than 5.

**Table 4:** Characteristics of participating practices

**Participating practices**	**Number of GPs in practice**	**ASGC-RA Remoteness of practice location**^a^	**SEIFA decile of practice location**^b^	**Resident population**
Practice 1	≥ 6	RA2	2	4,668
Practice 2	≤ 5	RA2	2	3,151
Practice 3	≤ 5	RA1	6	12,187
Practice 4	≥ 6	RA2	2	22,419
Practice 5	≥ 6	RA2	1	9,193
Practice 6	≤ 5	RA1	4	18,442
Practice 7	≥ 6	RA1	1	6,449

^a^ The Australian Standard Geographical Classification for Remoteness Area (ASGC-RA), with RA1 indicating a major city and RA2 an inner regional area [47]

^b^ The SEIFA decile is based on the Index of Relative Socio-economic Disadvantage (IRSD) and describes the decile ranking of the participating practices, with a decile of 1 indicating areas of most disadvantage and 10 indicating areas of least disadvantage [46]

Our integrated analysis of semi-structured interviews, participant observations and aggregate practice data is examined through the lens of the logic model with its domains of current environment, inputs, activities, outputs and outcomes/impacts (see Fig. 1). Refer to Additional file 1 for the full logic model.

### Current environment

The current environment domain of the logic model refers to the assessment of practice and system readiness for change. Key findings from the interview and observational data include, perceived need for change, team cohesion and centredness, meaningful use of data, referral pathways, culture of collaboration, and aware and motivated patients.

#### Willingness to undertake PCMH change

All seven practices perceived a need for change and developed their PCMH-based projects around this need, with the key driver being “to help improve health outcomes”:

…to actually develop a meaningful service for our small rural communities that has an impact in individual's lives, that will change the course of their life forever. (P1GP)

In P7, staff identified a need to create a coordinated approach to diabetes care management through review appointments. This would enable patients to receive more comprehensive care and education in managing their diabetes. P5 staff recognised a need to improve health literacy in terms of their patients’ knowledge and understanding about asthma/COPD management. A key motivator for P1 and P2 was the opportunity to increase teen access to mental health services, which were not readily accessible in their rural/remote towns:

We're hoping to increase the access, that young people in our towns will have to timely, free, quality health care, and that they can present for any issue. (P1PN)

In P6 staff perceived that patient care could be enhanced through an in-house consultant pharmacist, particularly if patients were taking multiple medications:

The concept of having an in-house pharmacist overseeing medications and making sure that patients understand what they’re taking (including complementary therapies) … and interactions was very appealing to me. (P6GP)

P4 saw the need for health measures to aid bone health in preventing osteoporosis, and P3 perceived the need to support the psychosocial needs of refugee patients by implementing a holistic psychosocial model that could “empower” these patients suffering from distress and trauma:

We're looking at the holistic model of wellness from the inside out because all of these participants have had many levels of loss and grief through their trauma. (P3PM)

It was also important for practice staff that their PCMH projects were feasible, sustainable and transferable to other general practice settings:

…knowing whether it’s a practical model that can be both re-producible for us in terms of ongoing, but also, is it a model that could be embraced by other practices because it is simple enough, and practical enough, and a financial model that can work? (P5GP1)

For example, the goal of P1 was to introduce their Teen Clinic model into other practices in the region, particularly for small rural communities where it was difficult to attract funding; “to do it in a way that means it's reproducible in other small communities” (P1GP). Likewise, observations indicated that P4 was driven by the idea of developing a transferable model of bone health that could also be adapted to other health conditions. In both of these projects, all aspects of the project were monitored and a cost analysis of practice nurse time on the project was undertaken for the purpose of transferability to other practices. For P3, creating a transferable holistic psychosocial model was perceived as an important contribution to refugee health.

#### Capacity to undertake PCMH change

It was observed that practices either had existing infrastructure capacity (human and physical) to undertake their projects, or used project funding to assist them. The capacity to undertake change was assisted by the ability of practices being flexible, as well as by having a sense of team centredness and cohesion:

And the practice needs to, as they evolve, become a little bit flexible…it's really important that no one is turned away. Even if you can't deal with whatever they need that day, at least they need to be sort of triaged and booked in somewhere along the line. (P1PN)

We’ve actually also got the nurses on board...the reception girls are even suggesting to patients…would they like to have a chat with the pharmacist while they're waiting for [name of GP] or vice versa. (P6PM)

All practices were engaged in the meaningful use of data (using data to help improve care) to inform their practice. For some practices, this involved staff training and sharing of expertise. In P5, for example, the GP leader trained the practice nurse to use clinical audit software to identify the target patient population required of for their project, which in this case was patients living with Asthma/COPD:

One thing in particular I have achieved from this is learning how to look at a [clinical audit] tool - looking at demographics and learning how to actually extract the information I want. (P5PN)

The P5 practice nurse worked together with GPs in the practice to select suitable patients for the integrated respiratory clinic. This collaborative approach also helped to engage GPs in the shared care clinics.

#### Local health readiness and system enablers for PCMH change

Identifying appropriate pathways to referral services was reliant on practice staff networking with local services and engaging with their communities and local stakeholders:

Those referral pathways have to be super local...can’t be done by an overarching thing…it’s really important that it is really local and that they can make their Teen Clinic really relevant to them. (P1PN)

P1 staff noted that their level of integration with local health services prior to implementation of the Teen Clinic had been “minimal”. Staff began to identify champions who could promote the Teen Clinic and build a culture of collaboration and trust to engage with the wider community:

…it really, really relies on building a relationship and trust with other providers in the community. So, definitely the schools have to understand what it's about and trust your service. (P1PN)

The practice nurse leader in P2 promoted the Teen Clinic within local high schools which was perceived to be an important system enabler for the project:

…it’s really important that they should not only be aware of us, but also helpful in promoting it as a safe place for them to go to. I have met with the local high school welfare officer who is really supportive and how they can work with us and how we can get the information out to the school kids. (P2PN)

Likewise, in P5, building a collaborative approach with the Local Health District (LHD) was perceived as a way of “working together or closely or at least being consistent” (P5GP2) in providing patient education and awareness and encouraging motivation.

### Inputs

Inputs in the logic model refer to the targeted training and support for PCMH change that the seven practices received that was tailored to their individual project.

Findings from the interviews demonstrated that some practices had more experience with PCMH change when compared to others. For example, P1 had established processes and resources for the Teen Clinic prior to the commencement of their project.

All practices participated in leadership and change management training provided by the PHN, as part of their preparation for PCMH change. The PHN supported the development of the project plans and outcome measures through regular touch points with all the practices. They also helped link practices to resources and existing tools, providing information and helping set clear boundaries around what the practices aimed to achieve:

…just ensuring that there’s regular progress points along the way and that we identify the risks up front and that we’re managing the risks along the way. (PHN8)

…developing relationships with the practice…consistently checking in with them. We want to keep them engaged in the project. If they do come across any challenges then we want to be able to address them quickly. (PHN3)

This also involved being realistic about what could be achieved within the timeframe: “Anything that involves change takes time. You have to be persistent and tenacious” (PHN9).

Peer meetings of practice nurses in P1 helped build a support network among practices to share their experiences and identify solutions as a group. This network became a key support when developing resources for HealthPathways (a web-based information portal to help primary care clinicians make assessment, management and specialist request decisions on how patients are managed in the local community context). In P5, cross system relationships were developed with the practice nurse leader and respiratory Clinical Nurse Consultants (CNCs) in organising and conducting the respiratory clinics:

She’s [respiratory CNC] actually visited our practice here and I’ve visited hers…We touched base to see how we were going do the clinic, and how she wanted it organized. And then also we touched base to see what resources that we would require at the clinic to give to the people prior…and during the clinic. (P5PN)

In some practices, clinical champions demonstrated PCMH change management. For example, in P1 the practice nurse and practice manager were the supporting drivers for four other Teen Clinics. They used experiences and reflections about lessons learnt from their prior implementation to assist with training and mentoring staff in other GP practices:

So for us it would be a mentoring role to provide them with all the resources, and the education to talk about our experiences that we’ve had while we’ve had the clinic running for two years. (P1PN)

Some practices used financial support provided by the PHN to support training of practice staff or to increase the practice infrastructure capacity. For example, by modifying practice rooms to support patient group sessions, or renting a room for a consultant pharmacist two days a week:

The access to the funding which enabled us to make some room changes and the whiteboard and things like that certainly has impacted on the success of the project. Having the dedicated room made a big difference. (P3GP)

### Activities

Activities in the logic model refer to PHN activities to support practices in PCMH change and various endeavours to motivate practices and patients.

Findings from the interviews identified that all practices were engaged in various PHN programs prior to the PCMH project. This engagement continued during the implementation of the projects with the PHN facilitating access to information, training, education and support to help build capacity for PCMH change. This was supplemented with professional development days that the PHN facilitated for various staff roles, including practice managers and practice nurses. Twenty two practice nurses and 35 practice managers participated in these activities. The PHN staff assisted in linking up and providing the practice workforce with specific training. The PCMH project funding for example, supported local readiness for PCMH by contributing to practice nurse training and Medical Practice Assistant (MPA) training. Twenty administrative practice staff enrolled in the MPA course, including three receptionists in P7:

It's also empowering staff...we heard that three of them [the receptionists] will be trained up as medical assistants (PHN3).

Other activities the practices were involved with included PCMH project group sessions, HealthPathways development and My Health Record discussions (a national online patient controlled electronic health record that contains key patient health information such as allergies, medications taken, medical diagnoses, and pathology test results). Practices undertook quality improvement activities such as use of the Primary Care Practice Improvement Tool (PC-PIT); data cleansing and data-driven quality improvement. Professional development was also undertaken, including change management training; leadership courses; health coaching and motivational interviewing.

The promotion of the PCMH projects was seen by the PHN as an important aspect of their role; “sharing stories about what other practices…might be doing” (PHN7):

I think that there will certainly be opportunity to kind of promote this and make them an exemplar to their peers. (PHN6)

Various activities were used to promote the PCMH projects and engagement from patients who attended the different practices. For instance, P3 harnessed the local print media to raise awareness about their project. Whereas, P1 and P2 used school visits and visibility at community events to help promote their projects and to motivate patients to use the Teen Clinics:

I guess there’s been some events where we’ve offered our presence. Whether it’s Youth Week events or there was screening of movies in [name] where the [name of clinic] wanted to be there, so they just wanted some support. (P1PN)

The PHN monitored the progress of the projects to help ensure that deliverables were met. A status report template was developed to monitor project actions and potential risks.

### Outputs

Outputs in the logic model refer to the products of activities that build capacity for PCMH change, such as resources, programs and services. Findings from the interview and observational data showed that practice wide leadership, teamwork, benchmarking, and practice staff working to top of scope were key outcome facilitators, as were processes for engaging patients and implementing innovative ways to improve patient access.

#### Engaged and willing workforce to undertake PCMH change

Many examples of practices being engaged and willing to undertake PCMH change were noted, for example, practice staff from P1 visited the other Teen Clinics who were also part of the PCMH projects, prior to implementation to share their established processes and resources. In P7, the GP principal and practice nurse organised regular planning sessions prior to implementing the diabetes review appointment and developed a template to be used as a guide for the patient review:

There’s a list of things that I do, including a survey to start off with on what their ideas are about diabetes, their knowledge and also how they feel they’ve been looked after. I then go through measurements and sort out their blood form and all that kind of thing...Then the GP's focus is wholly and solely on their diabetic management. (P7PN)

Although staff were willing to undertake PCMH change, many participants found the amount of time required to implement their project challenging. For example, P2 found the set-up of the Teen Clinic, in terms of time expended, to be “quite overwhelming”. However, they did suggest that this pressure became less challenging after the initial set-up:

I think the initial set-up takes time but I really believe that once the initial set-up is done, the ongoing project…takes maybe a half an hour to prepare each session beforehand. (P4PN)

Even though some practice staff perceived that there was a lack of dedicated time for their projects, others, such as the practice nurse leader in P5, were given protected time, supported by PHN funding, to conduct specific project tasks.

Practices were involved in setting project goals which included establishing benchmarks to achieve data driven outcomes. P1 established a process to access and observe their practice data which could be implemented across all Teen Clinics*.* In P6, the pharmacist engaged the PHN to create a data collection tool enabling an audit of patient medication adherence and a record of medication management interventions. P7 set benchmark targets prior to the commencement of their project, including the number of patients with HbA1c above 7.0 mmol/L; the number of patients with an HbA1c recorded in their medical record in the last 6 months; and the number of patients with a coded Type 2 diabetes diagnosis.

#### Enhanced capacity to undertake PCMH change

Practice-wide leadership and support for the projects from all practice staff were major contributors to undertaking change; “Something that [has] got to run effectively and efficiently in a practice needs to have all team buy-in” (P5GP1). P1 had a supportive team structure which was led by the GP principal and was facilitated by a strong practice nurse and practice manager:

The nurses need to be 100% on board but they need one other really key person…If you have got the GPs on board it makes it a lot easier or even one key GP on board. (P1PN)

In P5, the practice nurse leader acted as the practice champion in leading the project; “We’ve got the nurse as the practice champion and she’s taken it over. We’ve been much more successful”(P5GP2). Conversely, a lack of continued engaged leadership was a barrier to undertaking change. Observations revealed that P2 found ongoing implementation challenging with staff feeling they needed to prove the worth of the project, both fiscally and via patient outcomes.

Interdisciplinary communication helped practices facilitate a team-based approach to the projects. It was observed in P6 that the co-location of the consultant pharmacist in the practice setting facilitated a working relationship with the GPs. Informal conversations helped build rapport and encourage patient referrals. Practice meetings were also used to educate staff about the projects:

We have a great responsibility for education, so we believe in setting aside dedicated time to educate GPs in these sorts of things. (P4PM)

At P7, at the commencement of the project the practice nurse and lead GP met weekly. This dedicated time expedited the integration of the project into the everyday practice workflow and helped to establish clear roles:

We had to actually say, “Okay, when the person comes in and sees you for the first time, what do you actually need to check and achieve? How are we going to enter that into our computer system, so that that information is readily accessible by the GP when the GP comes to review the patient?” So we had to spend quite a bit of time sitting down and talking about that…to ascertain how that was actually going to work. (P7GP)

Patient engagement was a key component of enhancing capacity to undertake PCMH change. All practices used a variety of methods to engage with patients. In P7, the practice nurse played a key role in patient engagement with nurse-led diabetes review appointments and provision of information about multidisciplinary health care providers who could assist patients with their diabetes management. In P6, the pharmacist used waiting room advertisements, reviewed appointment lists and targeted patients, as well as actively reminded GPs to refer patients:

I actually realised the best thing to do is to call patients who are coming in on the day where I'm here, and try and team up for them to see me...that’s worked really well. (P6PH)

In P3, one-on-one sessions with the social worker, group psychosocial educations sessions, patient health journals, and a WhatsApp group were used to engage with patients. Being understanding and flexible was also important in terms of building patient’s confidence and trust in the project:

We also have to be a little bit flexible...the first session ran over because people came late, and that was partly because some of them forgot. (P3PM)

The Teen Clinics engaged with the wider community through practice nurses establishing relationships with support networks in the community, including local high schools, and by raising awareness at community events.

There are many examples of practice staff working to top of scope, as well as upskilling and multiskilling. In P7, the practice nurse took “a two day course in Sydney that was just completely about diabetes” (P7PN). She also learned how to use the patient billing system, underwent clinical auditing training, and a motivational interviewing course to assist with motivating patients about behaviour change. In P5, the practice nurse leader took additional asthma and COPD training:

There was a lot of professional development for me particularly…I find that there was improvement in my knowledge, particularly in looking at the stepwise management of COPD and what standards that they use. (P5PN)

The three receptionists from P4 that undertook MPA training in order to support Osteoporosis patients extended their roles by assisting the practice nurse with taking patients’ weight and height, bagging urine samples and entering notes into patient files, rather than being “just on reception answering phones”. Practice nurses in the Teen Clinics engaged in teen health training and the practice nurse leader in P4 attended Osteoporosis training. Working at the top of their licence, upskilling and multiskilling were reported to increase job satisfaction:

…getting the positive feedback from the patients that gives me a lot of satisfaction. To see them actually talking about implementing different things that we've talked about, to see their engagement in the actual sessions has been really, really satisfying. (P4PN)

#### Enhanced local health readiness for PCMH

Some projects were instrumental in increasing the integration and coordination of health services. P4 for example, built stronger multidisciplinary networks through consultations with their local radiology clinic to coordinate care. Recorded data for this project showed that twenty-one patients from the first recruitment round went directly to the radiology clinic after their bone health group sessions. In addition, Residential Aged Care Facility (RACF) staff were invited to participate in subsequent bone health sessions, providing the opportunity to share and transfer bone health prevention activities in the aged care setting:

So the plan for them is to then go back to the nursing home and while we’ve got one patient in mind, they’ll go back to the nursing home and use the exercise and diet tips on all of the patients. (P4PM)

It was noted that stronger relationships were also built between the practice staff and allied health providers (dietician and exercise physiologist), where previously there had not been the opportunity to meet the GPs that referred patients.

P5 built on existing relationships with the LHD, ensuring better collaboration and referral processes. The practice nurse leader collaborated with the respiratory CNCs to determine clear roles in the shared care clinics and to source relevant asthma and COPD resources:

We know when they go to see [name], what she will have covered and know what we can say to reinforce those messages, instead of adding confusion. (P5GP1)

#### Process to incrementally build system capacity for PCMH change

It was observed that P1 staff actively engaged with improving patient access through various innovations, including their practice website and a Teen Clinic Facebook page. A promotional video was developed and embedded in the webpage, and an ‘app’ developed which could be downloaded onto mobile phones to book appointments. In P3, the WhatsApp group was demonstrated to be an acceptable format for patient engagement:

…the first difficulties we had initially in terms of communicating and getting everyone to open up. We overcame that by creating a WhatsApp group...all of the participants, we chat between us. (P3PM)

Several of the projects were identified as exemplar models. P1 was invited to speak at PHN-led professional development days for practice staff and at the Australian Practice Nurse Association (APNA) conference in Brisbane in May 2018. The Teen Clinic model was also highlighted as a case study example on the APNA website. Likewise, the Bone Health PCMH project was identified as an exemplar model for chronic disease prevention in general practice and project staff also presented their project at the APNA Conference.

### Outcomes

The outcomes domain in the logic model refers to the evaluation and consolidation of PCMH change. Key findings from the interviews, observations and aggregate practice data include defined goals for PCMH change, review within practices, effective team function, data used to define quality targets and measure outcomes, practices engaged in supportive peer-networks, patients engaged in own care, coordinated and integrated care enhanced, and community awareness of PCMH change. Long term outcomes and impacts will require a longer duration before findings can be reported.

#### Motivated practice teams working towards agreed goals

It was observed that project teams worked together towards defined PCMH goals. In P7, the practice manager also noticed more diabetes care awareness with GPs in the practice:

I think the doctors are more aware of encouraging the patients to come see [name of practice nurse] for assessments. I think it's increased the doctors looking at their patients with the condition. (P7PM)

The practice manager in P4 described how they were keen to further integrate the MPA role into the coordination of patient care:

So our longer term plan is for the MPAs to take a section of the alphabet so to speak and look after that group of patients, so the patients know that that’s their go-to person. (P4PM)

In some practices it was noted that it took several weeks for a cycle of change to be established, for example, for the consultant pharmacist in P6 to receive referrals from the GPs in the practice. However, frequent reminders by the practice manager and pharmacist aided this change.

All practices were involved with reviewing their projects. P1 staff held a reflective workshop with other Teen Clinic staff to reflect on challenges and successes. They also conducted an internal review of their Teen Clinic with findings revealing that despite some initial stress with implementation, staff found the Teen Clinic a rewarding and positive experience. The trained MPAs in P4 provided positive feedback; “they’ve really enjoyed the project” (P4PN).

Interviewees perceived however, that the limited time to run their projects impacted on opportunities for review and reflection:

The other issue was this quite short timeline for this particular project so that you couldn’t refine and then redo the clinics to try and see whether something worked a little better. (P5PN)

Change fatigue was observed to be a challenge experienced across some practices, with practice nurses in particular often working long hours and on weekends to help manage change stress. Some staff reflected on additional training support that could be useful in future project iterations, including training in project management and budgets:

[It’s] one thing to get the grant money but then it’s knowing what to do with it, or how to keep track of it - the budget and accounting side. (P1GP)

#### Adaptive reserve within practices enhanced

Regular team meetings among practice staff helped to support team function and ensure whole team buy-in:

Weekly practice meetings... it's a good spot for [the practice nurse] to come up and tell us how she’s going for recruiting people…or to say “We thought the clinic ran a bit tight last time, we might need some more time on the next one.” (P5GP1)

Team meetings allowed staff to meet and reflect on processes and issues and to identify what could be changed to improve the projects; “what can we do better, looking at the resources and the translations of them, picking up any mistakes” (P3PM). Regular meetings with the PHN were also useful for motivating practices and keeping the projects on track:

[The PHN] has been really good. Our regular catch up meetings have been great because it’s helped me reflect as well on the project goals and be mindful of the limits. (P4GP)

Establishing local relationships with services outside the practices enhanced comprehensiveness of care with two way communication channels and referral pathways. Communication within peer networks enabled staff to engage in shared learning.

It was observed in many of the practices that the projects facilitated patients to become more engaged in their own care. In P3, fifteen participants (6 males and 9 females) took part in the self-care for refugee trauma project. There was high attendance and engagement with the seven psychosocial education sessions and up to 12 participants participated in each of the 19 walking sessions. As a result, participants developed 90 bi-lingual cards based on their own experiences to be used in future sessions to promote hope, strength and resilience. A GP in P6 reflected that patient engagement with the consultant pharmacist improved patient knowledge and understanding about their medication:

I think [it] has a profound effect on them feeling like they’re a part of their care, their understanding of their condition, their compliance with their medications…I think it’s a part of making sure that patients feel a part and somewhat in control of their own health. (P6GP)

Patients who participated in the Bone Health PCMH project in P4 were observed to be engaged in setting bone health goals and completing action plan diaries. The education sessions that the practice nurse facilitated were perceived to provide a supportive atmosphere:

We’ve got a group of patients out there who are now more aware of the dangers and how to avoid them so for proactive health and for health prevention that’s great. (P4PM)

GPs in P5 noted that patients in the respiratory clinics had become more engaged in their COPD management:

I’ve got the summary from the last COPD clinic, and their feedback was that they know that they weren’t using their medications correctly, they feel more confident that they understand the action plans, they’re planning to quit smoking… (P5GP1)

All practices collected aggregate data to measure project outcomes. For example, in P4, where there were targeted Bone Health sessions over a 6 month period (November 2017-May 2018), bone density screening increased from 9.6% to 26.95% for patients aged 70–79 years. Coding of Osteoporosis and Osteopenia in patient electronic records also increased and 10 new care plans were put in place to support newly diagnosed patients. Twenty-one patients attended group sessions and 17 set bone health goals and completed diary action plans. The pharmacist in P6 consulted with 140 patients between 10 January 2018 and 6 June 2018. The data revealed that 125 patients (89.2%) were taking more than five medications. Patients also commonly reported taking fewer medications than reported in their electronic health record. Patient records were updated and referrals were made to the care plan nurse. One hundred and twenty-seven patients were recalled for a medication review. Data collected from the Teen Clinics from 6 February to 6 June 2018, showed the majority of patients were female (*n* = 80) with mental health support (*n* = 31) and sexual health support (*n* = 45) the most common reasons for attendance. In P3 the fifteen patients in the self-care for refugee trauma project improved in the mean scores of the DASS 21 survey [48] over a five month period in each domain of depression; anxiety and stress. Likewise, results for the K10 survey [49], a measure of psychological distress, also showed an improvement in the mean scores of the 15 patients. In P5 seven patients participated in the asthma clinic (age range 22–55) and five in the COPD clinic (age range 66–77) and in P7 seven patients either participated or were booked into a diabetes clinic.

#### Local health system receptiveness to PCMH

The Teen Clinics engaged with local social and health services to help integrate and coordinate teen care services in the community; “I’ve seen an increase in the communication with the other providers on the outside” (P1PM). Local health provider attitudes changed toward the asthma/COPD clinics in P5 with the LHD offering additional “train the trainer” education sessions on asthma and COPD. As a result the GPs in the practice adjusted the action plans they provided to their patients:

We have got better action plans so patients can self-manage better and we’re using those more flexibly for patients, basically because [CNC 1] and [CNC 2] as well have both shown us more ways of customising those and choosing which version for which patient. (P5GP1)

#### Priorities for system-wide enablers

Many of the practices prioritised making the community aware of their PCMH activities, for example, through involving RACF’s in bone health sessions, and relationship building with local schools and support services. Data collected from the Teen Clinics for four months showed that 112 patients were seen over the five practices with 65 recorded initial visits and 47 return visits. Staff in P1 surveyed patients to investigate how and why teens engaged with the clinic. In P2, seven teenage patients returned to the Clinic for support out of the 31 who accessed the service. A longer period however is required to observe the Teen Clinic effectiveness. Similarly, the sustainability of all the projects will be a gauge to their effectiveness; “If that then could be scaled up and given to other practices across the region” (PHN2):

I would judge it successful if there’s a robust model delivered… this model could be put into practice somewhere else. So, I think that would be a success for me. (PHN4)

## Discussion

Our findings provide insights into the experiences of implementing PCMH change in seven Australian general practices. Through PCMH-related projects, each practice focussed on an area they perceived needed to change to become more aligned with PCMH goals. Throughout implementation of the projects it is evidenced that adaptive reserve within practices was enhanced; practices engaged with multidisciplinary teams, peer networks, patients and the wider community. There was an increase in data informed practice, patient engagement and motivation, and enhancement of staff roles through upskilling and multiskilling. It was evidenced that all seven practices were in the process of implementing the building blocks of high-performing primary care, particularly foundational elements of engaged leadership, data driven improvement and team-based care [16]. To the knowledge of the authors, this is one of very few systematic descriptions of explicitly PCMH-related change in Australia. The study contributes to the literature by demonstrating that international experience, combined with local consultation, can be successfully used to implement PCMH-related change across national contexts.

Our findings, under the lens of the logic model, demonstrate that PCMH change efforts in Australian general practice require willingness, capacity, local health readiness and system enablers for effective implementation. Although all practices were willing and motivated to undertake PCMH change, their capacity to undertake change differed. Some practices were more ‘local health ready’ for PCMH change compared with others and had system enablers in place. For example, P1 had prior experience in developing PCMH-related projects and had advanced processes for building patient engagement and community awareness, while other practices developed their projects de novo, which was time consuming for staff, particularly at the initial stages.

Key facilitators in the implementation of the projects included motivation for change, engaged leadership and strong change teams, as has been described in other international studies reporting on PCMH change [14, 18, 35, 50, 51]. Core change teams were versatile in staff composition, consisting of a GP leader to drive change within the practice team or a practice staff member as the driver, with GP support. This diversity in change leadership indicates that the logic model could be applied over a variety of practice sizes, geographic and socio-economic contexts and clinical conditions. Conversely, a lack of continued engaged leadership was cited as a barrier to PCMH change.

Consistent with other studies our findings indicate that communication between all key project players is crucial for successful project implementation. Routine meetings are important to discuss practice change and to ensure whole team buy-in to the projects [14, 52]. Ongoing communication and relationship building with allied health services and LHD staff is important for service integration [53]. Interviewees reported enhanced job satisfaction through working as part of a team and through opportunities to work to top of scope. These factors have been evidenced to improve sense of achievement and professional and personal growth [54].

The practices demonstrated that positive movement towards the PCMH foundational building blocks within a short period of time is achievable with PHN support. Interviewees highlighted the support provided by the PHN, including training, tailored practice visits, and funding to improve infrastructure and physical space, as being valuable to enhancing their capacity for PCMH change. This key role of the PHN in supporting PCMH change highlights the value of PHNs in Australia being funded to support local health care innovations [6, 11]. It also exemplifies the importance of supporting change towards a PCMH approach, whether small scale projects or full PCMH transformation, despite the continuing need for funding reform at a systems level.

The time required to implement the projects was a challenge in all practices. Time to set-up processes, develop workflow plans and reflect on the implementation progress required significant commitments and a substantial amount of in-kind work by practice staff. This barrier to change is a common finding in other studies [35, 52] and highlights the need for protected time to effectively bring about change and maintain staff satisfaction as well as continued support to alleviate change fatigue.

There were concerns regarding the viability of the projects post-funding. A twelve month review from the PHN to assess the progress and effectiveness of the projects was included as part of their service agreement. This will be critical to understanding the sustainability of the projects, and along with the logic model will assist in measuring longer term outcomes.

### Limitations of the research

The implementation of the projects remains a work in progress in the seven general practice sites. Our findings therefore may not present a complete picture of the implementation experience and as outcomes of the PCMH model in Australia are not yet known, an evaluation cannot be contextualised. Experiences may be different for other practice types. Future research and evaluation should include a range of practice types and in different geographical and socio-economic settings as well as incorporate patient and community input into the design, planning and implementation processes.

## Conclusion

Implementing change towards PCMH goals in Australian general practice is potentially achievable in a relatively short time-frame with PHN support, but requires strong leadership to promote a shared vision and purpose, and to engage practice staff and the wider community. Implementation of such initiatives, nationally and internationally, requires dedicated time, infrastructure, as well as training and tailored support. Continued tailoring of support and monitoring of activities may improve practice engagement in PCMH change that aims to achieve more effective health service delivery and improved health outcomes. Longer term monitoring and evaluation will help gauge the sustainability of such initiatives.

## Supplementary Information

**Additional file 1**. Logic model developed for PCMH-related practice redesign.

**Additional file 2**. Interview guides.

**Additional file 3**. Practice audit tools.

## Data Availability

Raw files are not available due to privacy reasons. Findings are reported in the paper in the form of quotations. The full logic model and audit tools are included as supplementary files. For further details on the data contact Christine Metusela: metusela@uow.edu.au.

## Abbreviations

CNC: Clinical Nurse Consultant
COPD: Chronic Obstructive Pulmonary Disease
EHR: Electronic Health Record
GP: General Practitioner
HCH: Health Care Homes
LHD: Local Health District
MPA: Medical Practice Assistant
PCMH: Patient Centred Medical Home
PHN: Primary Health Network
RACF: Residential Aged Care Facility
RACGP: Royal Australian College of General Practitioners
