# Supplementary material for: Implementation of a patient centred medical home (PCMH) initiative in general practices in New South Wales, Australia
Source: BMC Fam Pract. 2021 Jun 21;22:120. doi: 10.1186/s12875-021-01485-x (PMC8215740; doi:10.1186/s12875-021-01485-x)
Supplement: Supplementary file 1 — Additional file 1. Logic model developed for PCMH-related practice redesign. [file 12875_2021_1485_MOESM1_ESM.docx]

## Additional file 1. Logic Model developed for Patient Centred Medical Home projects

| **CURRENT ENVIRONMENT** |  | **INPUTS** |  | **ACTIVITIES** |  | **OUTPUTS** |  | **OUTCOMES** |  | **IMPACTS** |
| --- | --- | --- | --- | --- | --- | --- | --- | --- | --- | --- |
| **Assessment of practice and system readiness for change** |  | **Tailored support for practice and system change through** **SENSW PHN** |  | **Current and proposed** **SENSW PHN activities to support change** |  | **Suite of tools and resources at practice and system level to facilitate change** |  | **Evaluation and consolidation of change, planning for next cycle of change** |  | - PCMH change support in SENSW PHN business plans and culture - Clearly defined pathways for PCMH change for practices - Clearly identifiable support services for practices - Establishment of vibrant communities of practice in PCMH change among GP practices across the PHN - Practices and patients are in control of change - Practices and patients are empowered for change - Practices and patients benefit from change |
| **Willingness to undertake PCMH change:**   - Perceived need for change - Satisfaction with aspects of current model of care - Anticipated impact on patient outcomes - Team cohesion - Financial viability of alternative models of care - Individual values and goals |  | **Accurate information to support need for PCMH change:**   - Clinical champions to demonstrate successful PCMH change management and outcomes - Support for practices to use outcomes data for benchmarking - Leadership and team effectiveness benchmarking - Examples of PCMH financial models - Values clarification to guide change |  | **Provide and facilitate access to information**  **for PCMH change:**   - Set up forums and communities of interest - Timely updates of SENSW PCMH and Commonwealth HCH - Exemplar practice stories shared - Clinical data auditing (Sentinel Practices Data Sourcing project) - Primary care practice improvement tool (PC-PIT) - GP cluster meetings and practice meetings - Financial modelling education |  | **Engaged and willing workforce to undertake PCMH change:**   - Education, practice support and web based resources provide knowledge and skills that promote evidenced based care - Processes and resources that develop knowledge and build practice capacity for PCMH change - Data driven outcomes and benchmarking - Further developed professional networks for GPs, PNs, PMs and AHPs |  | **Motivated practice teams working towards agreed goals:**   - Defined goals for PCMH change - Established cycles of change - Review within practices - Change fatigue (stress) mitigation strategies in place   *10 Building Blocks: Engaged leadership (1); Empanelment (3); Team based care (4);* |  |  |
| **Capacity to undertake PCMH change**:   - Leadership capacity - Practice management capacity for change management - Team-centeredness - Adaptive reserve - Comprehensiveness and continuity of care capacity - Meaningful use of data - Level of patient engagement in care |  | **Leadership and change management training:**   - Leadership training - Change management training and facilitation - Practice communication training - Practice systems (teams, access and coordination) development support - Peer support networks - Meaningful data use support - Patient education support |  | **Training, education and support to build capacity for PCMH change:**   - GP leadership training - Change management, training & mentoring - Practice communications training - Medical assistant training - Primary care practice improvement tool (PC-PIT) - Patient Reported Measures - Clinical data auditing (Sentinel Practices Data Sourcing project) - Collaborative relationships with SENSW PHN - Training in patient activation / motivational interviewing / health coaching |  | **Enhanced capacity aligned with and to undertake PCMH change:**   - Practice wide leadership - Robust change management plan - Inter-disciplinary communication - Data driven care - Enhanced use of shared electronic records - Practice staff working to top of scope - Medical assistants, clinical pharmacists and AHP in core team - Practice processes that promote data quality and systematic and planned care - Practice processes for engaging patients |  | **Adaptive reserve within practices enhanced:**   - Facilitative leadership and management models - Effective team function established in practices to ensure continuity and comprehensiveness of care - Data used to define quality targets and measure outcomes - Practices engaged in supportive peer-networks - Patients engaged in own care   *10 Building Blocks: Data-driven improvement (2); Team-based care (4); Patient-team partnership (5); Population management (6); Continuity of care (7); Comprehensiveness and care coordination (9)* |  |  |
| **Local health readiness for PCMH:**   - Integration and coordination of care - Available resources - Referral pathways - Communication (modes/effectiveness) - Relationships - Culture of collaboration |  | **Targeted support for integrating local health system:**   - Development and promotion of HealthPathways for common/ resource intensive CDM - Cross system peer meetings - Cross system relationship development |  | **Support local readiness for PCMH:**   - Ongoing collaboration with LHD - Secure messaging project - Develop practice and consumer resources re PCMH - Targeted HealthPathways development - Facilitate cross system peer meetings and online forums |  | **Enhanced local health readiness for PCMH:**   - Increase in integrated, coordinated health services - Up to date website providing practice info, resources and tools, and regular distribution of newsletters - Support that promotes technical innovation for communication across the health sector e.g. secure messaging - Multidisciplinary professional networks explored if not developed |  | **Local health system receptiveness to PCMH [Population management]:**   - Coordinated and integrated care enhanced   *10 Building Blocks: Population management (6); Prompt access to care (8); Comprehensiveness and care coordination (9)* |  |  |
| **System enablers for PCMH change:**   - Aware and motivated patients - Shared electronic records - Effective IT systems - Sources of accurate information regarding PCMH |  | **Local support for system enablers:**   - Patient and community awareness-raising and education - Shared electronic health records support - Advice re IT systems - Education / communication portals - Information clearing house |  | **Incrementally build system enablers for PCMH change:**   - Consumer health panel - Practice training for consumer engagement - Development of consumer resources - Practice training for patient activation - eHealth support for MyHR and secure messaging - Targeted HealthPathways development - Develop peer support networks and communities of interest |  | **Process to incrementally build system capacity for PCMH change:**   - Practice processes for patient engagement and activation - Practice processes for meaningful use of MyHR - Support that promotes technical innovation to improve patient access e.g. phone app and telehealth service - Up to date website providing PCMH practice info, resources and tools, and regular distribution of newsletters - HealthPathways aligned with CDM management - Exemplar practices Identified for peer support / mentoring |  | **Priorities for system-wide enablers:**   - Community awareness of PCMH change - Enhanced use of shared electronic records   *10 Building Blocks: Patient-team partnership (5); Continuity of care (7); Comprehensiveness and care coordination (9)* |  |  |
