# Supplementary material for: Implementation of a patient centred medical home (PCMH) initiative in general practices in New South Wales, Australia
Source: BMC Fam Pract. 2021 Jun 21;22:120. doi: 10.1186/s12875-021-01485-x (PMC8215740; doi:10.1186/s12875-021-01485-x)
Supplement: Supplementary file 2 — Additional file 2. Interview guides. [file 12875_2021_1485_MOESM2_ESM.docx]

## Additional file 2. Interview guides

**Pre interview guide for participating practices**

- *Firstly, I am interested to know what your involvement in patient centred medical home consultation has been to this point.*

Prompts: Were you involved in the consultation of PCMH? What have you heard? What education sessions have you attended?

- *In your experience, how could the types of models of care for the management of chronic disease in general practice, in which you have been involved, be improved?*

Prompts: GPs, practice staff, allied health: More structure; more self-directed; level of clinical contact; time demands

- *This evaluation is reviewing the Innovation Projects for the management of chronic disease in your general practice*. *Can you tell me about any experience you have had with patient-centred care in chronic disease management in the past?*
- *Tell me about the Innovation Project in your practice. What are you hoping to achieve? How are you hoping to achieve that?*

Prompts: Benefits, drawbacks, costs, technical issues, infrastructure issues, scheduling

- *What do you think would make your project a successful experience medically?*
- *How would you judge whether it was successful or not?*
- *What would you see as being barriers to success?*
- *What would you see as helping success?*
- *How do you envision the PHN will assist in the success of the project?*

**Post interview guide for participating practices**

- *Firstly, I am interested to know what your involvement in the Innovation project has been. How have you developed professionally from your involvement in the Innovation Project?*

Prompts: Improved knowledge? Improved skills? Improvements in your day to day work?

- *This project has involved an evaluation reviewing the Innovation Projects for the management of chronic disease in your general practice. Tell me about the Innovation Project in your practice. Did you achieve what you were hoping to achieve? How did you achieve that?*

Prompts: Practice redesign; Leadership; Team function; Practice processes/data driven care; Communication with LHD/AHP/Community health; How did your practice team integrate other services into everyday practice?

- *What do you think made your project a successful experience medically?*

Prompts: Patient engagement/activation; Community awareness about project

- *How did you judge whether it was successful or not?*
- *Of what value was the innovation project initiative:* To practice staff? To patients? To your practice as a whole?
- *What would you see as being barriers to success?*
- *What would you see as helping success?*
- *How did the PHN assist in the success of the project?*

*Prompts: Did you require support from the PHN to achieve the change?* How often was support provided? What support or resources were provided? How adequate was this support for you? *Was it useful?* I*s further support needed?* *How satisfied are you with the support given? If you didn’t require support, could you tell us why?*

**Pre interview guide for PHN staff**

- *Tell me about the Innovation Projects that are being run in your cluster. What are you hoping will be achieved? How are you hoping to achieve that?*

*Prompts: Expectations/goals*

- *How have you used the logic model to guide the co-design/selection process of the Innovation Projects?*

Prompts: Was it useful? Did you ignore it?

- *How would you judge whether the innovation projects were successful or not?*

Prompts: What do you think are some important questions to ask in this evaluation?

- *What would you see as helping the success of the Innovation Projects?*

Prompts: Co-design process; projects and evaluation

- *How do you envision the PHN will assist in the success of the project?*
- *What would you see as being barriers to the Innovation Projects success?*

*Prompts:* shortened timeframes*;* costs*;* technical issues*;* infrastructure issues*:* scheduling

**Post interview guide for PHN staff**

- *Firstly, I am interested to know what your involvement in the Innovation project has been.*

Prompts:

1. Could you please describe your engagement in the Innovation projects?
2. What is your involvement with implementation of the project/s?
3. What about in terms of improving the project/s?
4. How satisfied are you with your involvement?
5. Could you please describe the IT support you provided to general practices?
6. How has this (TYPE) support been able to assist practices?
7. Are practice staff developing competencies with IT as a result of PHN assistance? How?
8. What IT challenges are practices presenting with?

- *Tell me about the Innovation Projects that are being run in your cluster. Did you achieve what you were hoping to achieve? How did you achieve that?*

Prompts: Practice redesign; Leadership; Team function; Practice processes/data driven care; Communication with LHD/AHP/Community health; Patient engagement; Community awareness; Shared electronic health records

- *How have you used the logic model to guide the development process of the Innovation Projects?*

Prompts: Was it useful? Did you ignore it?

1. Was it used as a guide to develop education and web based resources? How?
2. Was it used to guide a suite of resources and tools for PCMH support and communication around change in practice?
3. Did you identify exemplar practices Identified for peer support/mentoring?

- *How did you judge whether the Innovation Project was successful or not?*
- *What would you see as helping the success of the Innovation Projects?*
- *How do you envision the PHN assisted in the success of the project?*
- *What would you see as being the barriers to the Innovation Projects success?*

Prompts: shortened timeframes; costs; technical issues; infrastructure issues*;* scheduling
