# Supplementary material for: Implementation of a patient centred medical home (PCMH) initiative in general practices in New South Wales, Australia
Source: BMC Fam Pract. 2021 Jun 21;22:120. doi: 10.1186/s12875-021-01485-x (PMC8215740; doi:10.1186/s12875-021-01485-x)
Supplement: Supplementary file 3 — Additional file 3. Practice audit tools. [file 12875_2021_1485_MOESM3_ESM.docx]

## Additional file 3. Practice audit tools

**P1: Teen Clinic data summary from cloud-based data collection tool**

| **Site summary** | **1** | **2** | **3** | **4** | **5** | **Total** |
| --- | --- | --- | --- | --- | --- | --- |
| Total number of attendees at each site |  |  |  |  |  |  |
| Age Range |  |  |  |  |  |  |
| Total number Male patients |  |  |  |  |  |  |
| Total number Female patients |  |  |  |  |  |  |
| Sex other |  |  |  |  |  |  |
| Number Initial visit |  |  |  |  |  |  |
| Number Return visit |  |  |  |  |  |  |
| Reason for presentation (list): |  |  |  |  |  |  |
| Number of patients who were a current patient at the practice |  |  |  |  |  |  |
| Number of patients not current patient at practice |  |  |  |  |  |  |
| Number of patients with a local GP |  |  |  |  |  |  |
| Number of patients referred back to local Dr |  |  |  |  |  |  |
| Number who saw nurse only |  |  |  |  |  |  |
| Number who saw Nurse and GP on the day |  |  |  |  |  |  |
| Referral other service provider |  |  |  |  |  |  |
| Number of patients who saw the GP at a later date (GP apt booked) |  |  |  |  |  |  |
| Other opportunistic screening that took place in a visit (list): |  |  |  |  |  |  |

**P1: Presenting teen patient knowledge source of Teen Clinic**

| **Site** | **Teacher** | **School Visit** | **Poster** | **Friend** | **Parent** | **Online** | **Unsure** | **Case worker** | **Practice staff** |
| --- | --- | --- | --- | --- | --- | --- | --- | --- | --- |
| **1** |  |  |  |  |  |  |  |  |  |
| **2** |  |  |  |  |  |  |  |  |  |
| **3** |  |  |  |  |  |  |  |  |  |
| **4** |  |  |  |  |  |  |  |  |  |
| **5** |  |  |  |  |  |  |  |  |  |
| **Total** |  |  |  |  |  |  |  |  |  |

P2: List of courses attended by practice nurses

| **Nurse identifier** | **Date** | **Facilitator** | **Title of course** | | **Notes** |
| --- | --- | --- | --- | --- | --- |
| **Courses attended** | | | | | |
|  |  |  | |  |  |
| **Course coming up** | | | | | |
|  |  |  | |  |  |
| **Future training** | | | | | |
|  |  |  | |  |  |

P3: Psychosocial education sessions participation numbers

| **Date** | **Title of education session** | **Total number of participants** | **No. Male** | **No. Female** |
| --- | --- | --- | --- | --- |
|  |  |  |  |  |

P3: DASS 21 survey results

| **Number who completed survey** | **No. Male** | **No. Female** | **Mean score start:** | **Mean score middle:** | **Mean score end** |
| --- | --- | --- | --- | --- | --- |
|  |  |  |  |  |  |

P3: K10 survey results

| **Number who completed survey** | **No. Male** | **No. Female** | **Mean score start:** | **Mean score middle:** | **Mean score end:** |
| --- | --- | --- | --- | --- | --- |
|  |  |  |  |  |  |

P3: Walking group participation numbers and comments

| **Instance** | **Date** | **Number of**  **Participants** | **Number Males/**  **Females** | **Comments by exercise physiologist** |
| --- | --- | --- | --- | --- |
|  |  |  |  |  |

P4: Project benchmarking data from the last 6 months of the Innovation Project

| **Activities** | **At start of project:** | **After 6 months:** | **Goal** |
| --- | --- | --- | --- |
|  |  |  |  |

P4: Patient recruitment methods and numbers

|  | **First & Second Group** | **Third Group** |
| --- | --- | --- |
| Number invited |  |  |
| SMS sent |  |  |
| Phone Calls |  |  |
| Booked |  |  |
| Responses |  |  |
| Interested – Try Again |  |  |
| Not Interested |  |  |

P5: Recruitment numbers

| **Clinic Type** | **Number of patients invited to the clinic** | **Number who participated** | **Cancellations** |
| --- | --- | --- | --- |
| Asthma Clinic |  |  |  |
| COPD Clinic |  |  |  |

P5: Demographics of shared care clinics

|  | **Number of participants** | **No. Male** | **No. Female** | **Age range** |
| --- | --- | --- | --- | --- |
| **Asthma Clinic** |  |  |  |  |
| **COPD Clinic** |  |  |  |  |

P5: Practice nurse time spent on project

| **Activity** | **Time spent on activity (in hours)** |
| --- | --- |
| Training |  |
| Resources |  |
| Meetings |  |
| Demographic |  |
| Patient Engagement |  |
| Clinic |  |
| Total |  |

P6: Summary data of pharmacist consultant intervention in general practice

| **Total number of patients involved in Pharmacist in GP review** | **Number** |
| --- | --- |
| Number of patients taking over 5 medications |  |
| Number of patients taking complementary medications |  |
| Average number of complementary medications taken |  |
| Number of patients with Diabetes |  |
| Number of patients with Asthma |  |
| Number of patients with COPD |  |
| Green card needed to be updated |  |
| Average number of medications being taken as per patients Best practice records |  |
| Average number of medications - patient self-reported |  |
| Medications being taken: |  |
| - Anticoagulants |  |
| - Antidepressants |  |
| - Antipsychotics |  |
| - Bladder antispasmodics |  |
| - Inhaled anticholinergics |  |
| - Injected hypoglycaemic |  |
| - Oral hypoglycaemic |  |
| - Respiratory medications |  |
| - Complementary medications |  |
| Average Drug Burden Index Score |  |
| Number of patients who had complementary medications added in the Pharmacist review conversation |  |
| Intervention provided: |  |
| - Drug list discrepancy |  |
| - Potential drug related problem identified |  |
| - Average number of drug interventions / interactions identified |  |
| - BGL self-monitoring counselling |  |
| - Diabetes and diet related advice |  |
| - Inhaler technique advice |  |
| - New glucometer and instructions provided |  |
| - Patient recalled for medication review and entered into BP |  |
| - New glucometer and related instructions provided |  |
| - Provide patients with medication list |  |

P7: Patient recruitment data

| **Diabetes Clinic Instance** | **Number of patients invited to the clinic (count)** | **Number who participated or booked clinic for future appointment (count/%)** | **Cancellations/declined participation** |
| --- | --- | --- | --- |
|  |  |  |  |
